# Seronegative secondary syphilis in a patient with hematologic malignancy on anti-CD20 therapy: case report and review of serologic responses to spirochetal infection in patients receiving B-cell-depleting therapies

**DOI:** 10.1128/asmcr.00010-26

**Published:** 2026-04-15

**Authors:** Sheikh Abdullah, Alexandre E. Malek, Elena Gonzalez Caldito, Ashley Flowers, Poornima Ramadas

**Affiliations:** 1 Department of Internal Medicine, LSU Health Shreveport 23346, Shreveport, Louisiana, USA; 2 Division of Infectious Diseases, LSU Health Shreveport 23346, Shreveport, Louisiana, USA; 3 Division of Dermatology, LSU Health Shreveport 23346, Shreveport, Louisiana, USA; 4 Division of Pathology, LSU Health Shreveport 23346, Shreveport, Louisiana, USA; 5 Division of Hematology and Oncology, LSU Health Shreveport 23346, Shreveport, Louisiana, USA; Rush University Medical Center, Chicago, Illinois, USA

**Keywords:** syphilis, obinutuzumab, anti-CD20-depleting agents, lymphoma, seronegative syphilis

## Abstract

**Background:**

The expanding use of B-cell-depleting anti-CD20 monoclonal antibodies has introduced diagnostic challenges for infections that are traditionally confirmed through serologic testing. Seronegative secondary syphilis is exceptionally rare and has historically been described only in profoundly immunocompromised patients with advanced HIV/AIDS. This case represents a novel clinical scenario in the era of targeted immunologic therapy.

**Case Summary:**

We report the case of a 44-year-old man with marginal zone lymphoma receiving maintenance obinutuzumab, who presented with a symmetric, pruritic, palmoplantar predominant papulosquamous eruption accompanied by fatigue and elevated inflammatory markers. Extensive serologic testing for syphilis, including repeated treponemal (TP-PA, treponemal IgG) and nontreponemal (RPR) assays, remained completely nonreactive on multiple occasions. However, a skin punch biopsy demonstrated epidermal hyperplasia with a dense plasma cell-rich lymphohistiocytic infiltrate. Immunohistochemical staining for *Treponema pallidum* revealed numerous spirochetes in the epidermis and superficial dermis, confirming the diagnosis of secondary syphilis. The patient received three weekly intramuscular doses of benzathine penicillin G (2.4 million units per dose) with complete clinical resolution and no adverse effects. Serologic tests remained nonreactive on follow-up evaluation.

**Conclusion:**

This case is the first documented report of persistent seronegative secondary syphilis in a patient receiving obinutuzumab therapy. It underscores the critical importance of maintaining a high index of clinical suspicion and pursuing tissue-based diagnostics when serologic tests fail to explain compelling clinical findings in patients with profound B-cell depletion. Clinicians managing patients on anti-CD20 therapies must recognize the limitations of serologic diagnosis for infections that typically depend on antibody detection.

## INTRODUCTION

Secondary syphilis is primarily a clinical diagnosis supported by serologic testing. However, humoral suppression from B-cell-depleting anti-CD20 therapies can blunt antibody production and yield false-negative treponemal and non-treponemal tests. Seronegative secondary syphilis has historically been described in profoundly immunocompromised states such as advanced HIV/AIDS. (1, 2) The growing use of anti-CD20 agents in hematologic and autoimmune conditions may compromise the reliability of serology-based diagnostic approaches in select patients. (3–5) We report a biopsy-proven case of secondary syphilis in a patient receiving maintenance obinutuzumab, whose treponemal and non-treponemal serologies remained persistently nonreactive, emphasizing the need for direct organism detection when clinical suspicion is high.

## CASE PRESENTATION

### Patient information

The patient was a 44-year-old man with stage III T-cell-rich B-cell lymphoma diagnosed in 2016, treated with six cycles of R-CHOP in 2017 with complete metabolic response and subsequent surveillance. In 2023, he underwent a relapse with biopsy-proven marginal zone lymphoma and received six 28-day cycles of bendamustine–obinutuzumab (bendamustine 90 mg/m² IV days 1–2 and obinutuzumab 1,000 mg IV per cycle) from July to December 2023, again achieving complete metabolic response. Maintenance obinutuzumab 1,000 mg IV every 8 weeks was started in January 2024 and continued through presentation, with the last infusion approximately 8 weeks before rash onset. He reported no prior sexually transmitted infections and denied intravenous drug use. Social history revealed unprotected sexual intercourse with a male partner approximately 8 weeks before symptom onset.

### Clinical findings

The patient presented with several weeks of progressive fatigue and a symmetric, intensely pruritic papulosquamous eruption that began on the trunk and spread to the extremities, palms, soles, and scalp. He denied fever, arthralgia, or pharyngitis. He also denied headache, visual changes, focal weakness or numbness, or other neurologic complaints.

**Fig 1:**
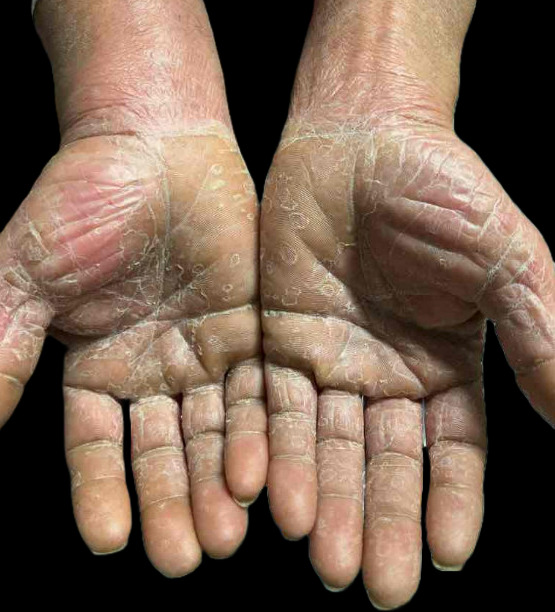
Palms showing diffuse desquamation intermixed with papules exhibiting the characteristic collarette of scale.

Physical examination revealed symmetric oval erythematous papules with a distinctive collarette of scale on palms and soles (Fig. 1). The trunk displayed thin, slightly hypopigmented papules on an erythematous background. There was no cervical or supraclavicular lymphadenopathy, and neurologic examination was unremarkable.

### Diagnostic assessment

Initial laboratory evaluation showed leukocytosis with a WBC of 13.57 × 10³/µL (reference 3.90–12.70 × 10³/µL) and neutrophil predominance, with an ANC of 8.48 K/µL (reference 1.8–7.7 K/µL). Hemoglobin was 7.6 g/dL (reference 12.5–16.3 g/dL), decreased from a baseline of approximately 9–11 g/dL. Inflammatory markers were elevated, including ESR 73 mm/hour (reference 0–15) and CRP 15.5 mg/L (reference ≤0.90). LDH was 218 U/L (reference 110–260). HIV-1/2 Ag/Ab was nonreactive, and CD4 count was 709 cells/µL (reference 401–1,532).

**Fig 2:**
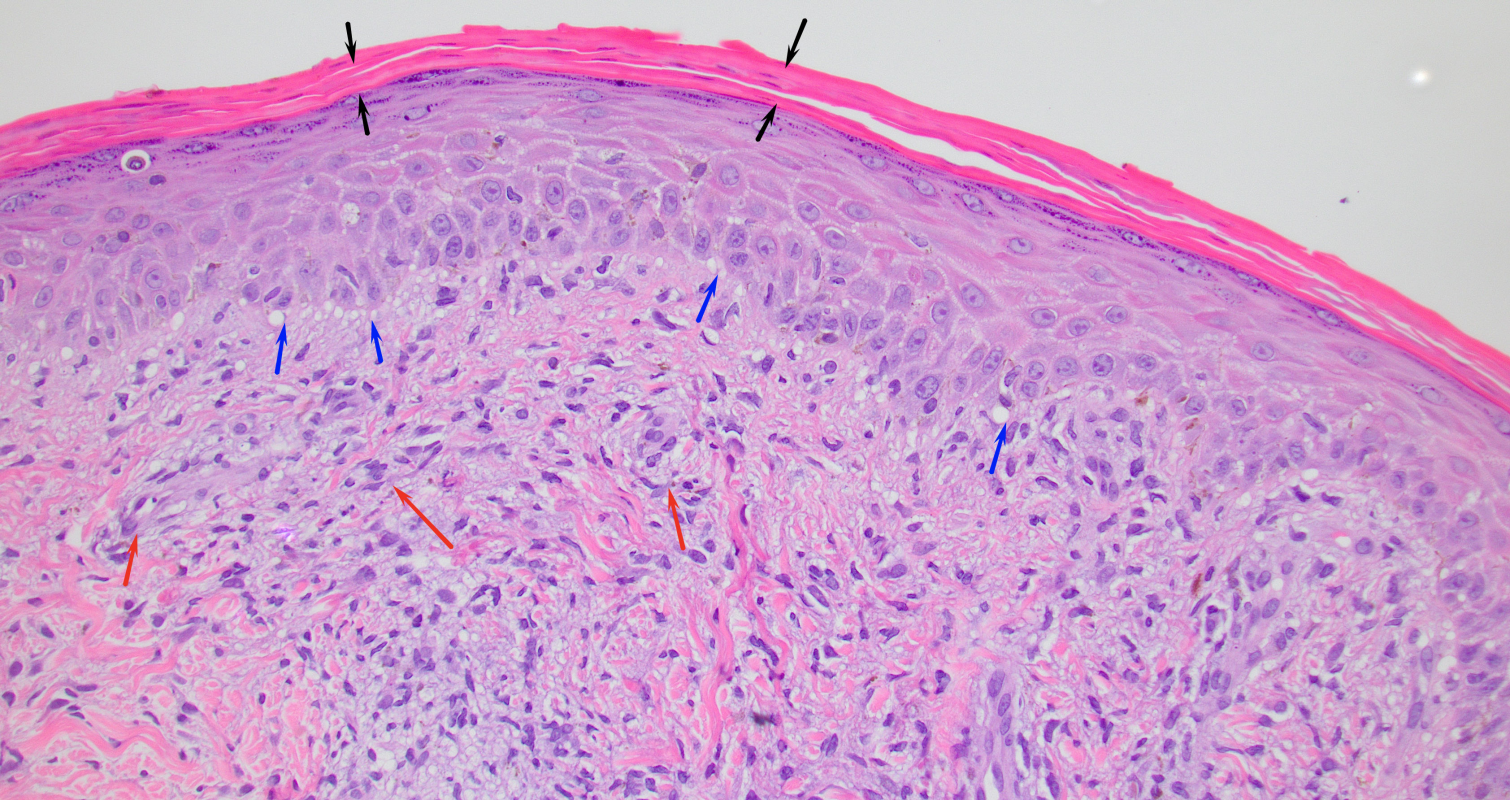
Punch biopsy showing parakeratosis (black arrow), vacuolar interface change (blue arrows), and superficial and perivascular lymphohistiocytic inflammation (red arrows). H&E 20×.

**Fig 3:**
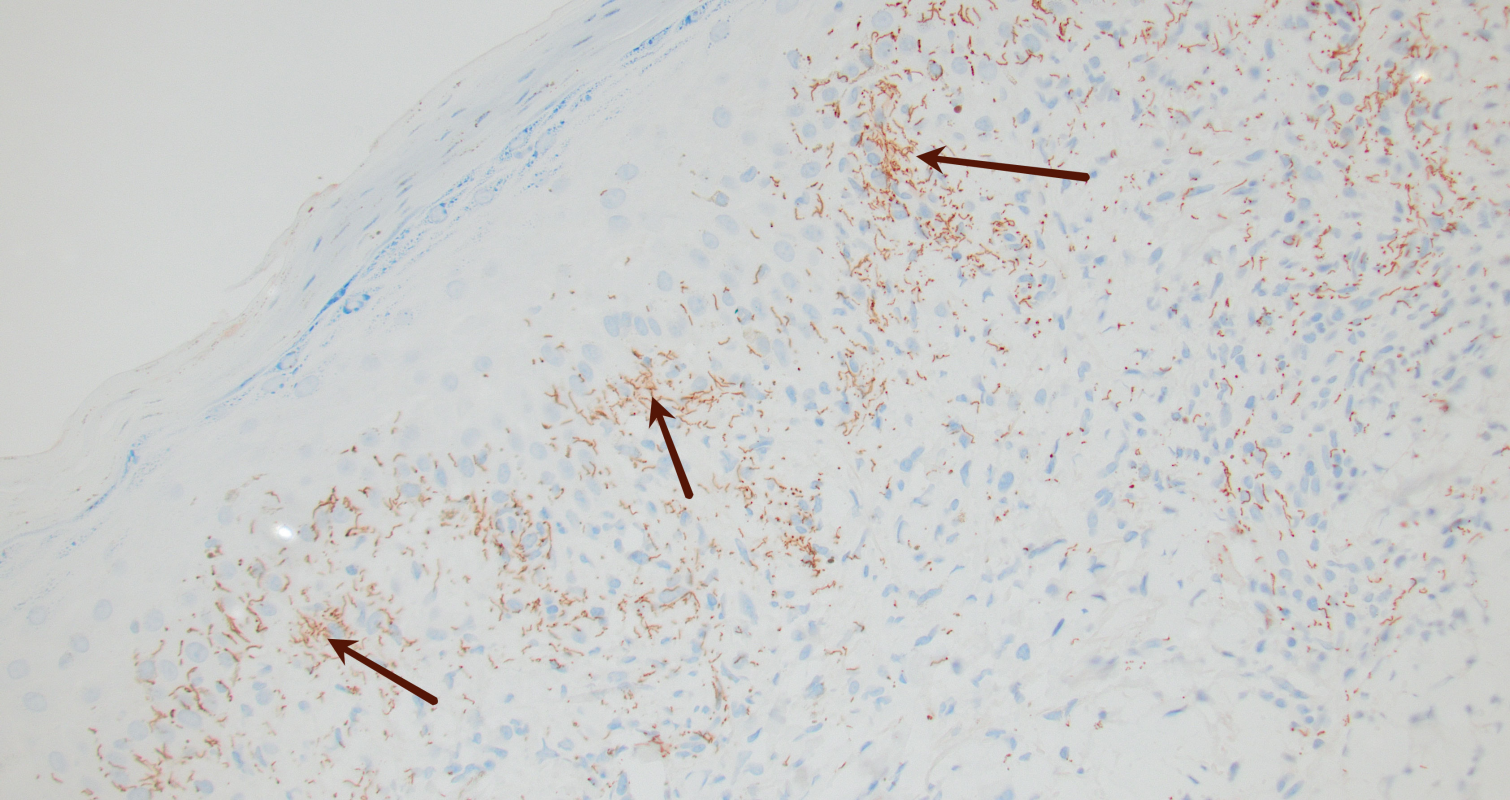
Spirochete immunohistochemical stain highlights numerous spirochete organisms (black arrows) within the epidermis and superficial dermis compatible with *Treponema pallidum* 20×.

HBsAg, HBc IgM, HAV IgM, HBsAb, and HCV Ab were nonreactive. CMV and EBV DNA PCR were negative. A respiratory viral PCR panel including *Mycoplasma* and *Chlamydophila* was negative, and blood cultures showed no growth. CT of the chest/abdomen/pelvis showed no occult infection or lymphadenopathy. Peripheral blood flow cytometry demonstrated a virtual absence of CD19+/CD20+ B cells, with lymphocytes composed predominantly of T cells, consistent with ongoing anti-CD20 therapy. Serum immunoglobulin levels were not obtained. At our institution, syphilis testing follows a reverse sequence approach beginning with a treponemal IgG assay. In this patient, the initial *Treponema pallidum* IgG screen was nonreactive; because clinical suspicion persisted, additional RPR and confirmatory TP-PA testing were also performed and remained nonreactive. A high-titer prozone phenomenon was excluded by repeat testing with dilution. A punch biopsy showed irregular epidermal hyperplasia with parakeratosis and a superficial/deep perivascular and interstitial lymphohistiocytic infiltrate (Fig. 2). PAS-D stain was negative for fungal organisms. Immunohistochemical stain for spirochetes highlighted numerous organisms in the epidermis and superficial dermis, confirming secondary syphilis (Fig. 3).

### Diagnostic challenges

The principal diagnostic challenge was the discordance between a classic clinicopathologic presentation of secondary syphilis and persistently negative treponemal and non-treponemal serologies in a patient with profound B-cell depletion from anti-CD20 therapy. Because serologic assays remained nonreactive, diagnosis required direct organism detection via biopsy and immunohistochemistry.

### Therapeutic intervention and follow-up

The patient received symptomatic topical corticosteroids for pruritus and definitive therapy with intramuscular benzathine penicillin G 2.4 million units weekly for three doses.

The patient tolerated therapy well and did not develop a Jarisch–Herxheimer reaction. On follow-up after completion of therapy, the rash had completely resolved. Repeat RPR remained nonreactive. Because serologic titers did not become positive, the treatment response was assessed clinically (resolution of symptoms and rash), with planned continued clinical follow-up. The patient was counseled regarding partner notification and treatment.

## DISCUSSION

We describe a biopsy-proven case of seronegative secondary syphilis (SNSS) in a patient receiving maintenance obinutuzumab for marginal-zone lymphoma, who remained completely seronegative on repeated non-treponemal and treponemal serologic tests. Seronegative secondary syphilis has been less commonly documented, typically during the very early window before antibodies develop or in profoundly immunocompromised states such as AIDS (1, 2). Our case is, to our knowledge, the first reported instance of persistent seronegativity in secondary syphilis under obinutuzumab therapy. In the contemporary era, B-cell-depleting anti-CD20 therapies introduce a new and increasingly relevant mechanism for seronegativity.

Anti-CD20 monoclonal antibodies (rituximab, ocrelizumab, and obinutuzumab) are widely used for hematologic malignancies and autoimmune diseases. These agents produce rapid and profound B-cell depletion; levels start to recover 6 to 9 months after therapy and may not normalize for a year (3). Hypogammaglobulinemia is a recognized consequence: in one study, approximately 39% of rituximab-treated lymphoma patients develop IgG <600 mg/dL, and 6.6% require IV immunoglobulin (4). Severe infection rates rise accordingly; one cohort of 4,479 patients noted an increase from 17.2% before rituximab to 21.7% after treatment, with a five-fold increase in infection-related mortality (5). Obinutuzumab achieves significantly deeper and more sustained B-cell depletion than rituximab through enhanced Fc-mediated effector functions (6). Clinical trials and pooled analyses have shown higher rates of grade 3/4 adverse events and infections with obinutuzumab-based regimens compared with rituximab. (7, 8) The deeper B-cell depletion likely explains why our patient never mounted detectable antibody responses. These immunologic consequences directly affect the accuracy of serologic testing, particularly for infections such as syphilis that rely on antibody detection.

Secondary syphilis is commonly diagnosed via a serologic response to infection. Non-treponemal tests (RPR and VDRL) demonstrate nearly 100% sensitivity and 96%–99% specificity in this stage, with confirmatory treponemal assays providing additional accuracy (9, 10). Since *Treponema pallidum* cannot be cultured, direct detection methods (PCR and histopathology) are usually reserved for situations where clinical suspicion is high despite negative serologic assessment (9, 10). While serologic assays are generally dependable, certain clinical scenarios have long challenged this assumption.

In the pre-antiretroviral era, severely immunocompromised AIDS patients occasionally manifested secondary syphilis with negative serologic tests (1, 2). Some early primary syphilis cases may yield false-negative results before seroconversion. A high-titer prozone effect can also cause negative non-treponemal tests, but dilution resolves that phenomenon (11). SNSS in an immunocompetent patient is extremely uncommon. More recently, with the advent of B-cell-depleting therapies, similar diagnostic pitfalls have re-emerged.

Several recent reports support the concept that anti-CD20 therapy can blunt serologic responses in syphilis. Lefeuvre et al. described the case of a 33-year-old man with multiple sclerosis on rituximab, who developed a classic secondary syphilis rash but had non-reactive VDRL and TPPA on repeated testing. Diagnosis was achieved only through a skin biopsy showing spirochetes via immunohistochemistry and PCR, and treponemal serology became weakly positive only after 3 weeks (12). Garbers et al. reported the case of a 22-year-old man on ocrelizumab who presented with an asymmetric, non-pruritic rash and systemic symptoms. His serum VDRL was only 1:2, cerebrospinal fluid VDRL was negative, and chemiluminescent treponemal assays were positive; he had low IgM (24 mg/dL) but normal IgA/IgG and negative HIV test (13). Pipitò et al. recently reported persistent negative treponemal and non-treponemal serologies in a patient on ofatumumab with syphilis and ocular involvement, with diagnosis confirmed by PCR (14). A summary of previously reported seronegative treponemal and other spirochetal infections under anti-CD20 therapy is provided in Table 1.

**TABLE 1:** Previously reported cases of seronegative treponemal and spirochetal infections in patients receiving anti-CD20 therapy^*a*^

Author, yr (ref.)	Patient and anti-CD20 therapy	Infectious agent	Serologic testing	Histopathology	Molecular/direct testing	Treatment and outcome	Key notes
Lefeuvre et al., 2021 (12)	33-year-old man with MS on rituximab	*Treponema pallidum* (secondary syphilis)	Repeated VDRL and TPPA negative; seroconversion only after >3 weeks	Skin biopsy showed plasma cell-rich dermatitis; Warthin–Starry stain positive for spirochetes	PCR and IHC for *T. pallidum* positive	Single-dose benzathine penicillin G; patient improved	B-cell depletion blunted antibody response; biopsy and PCR necessary
Garbers et al., 2024 (13)	22-year-old man on ocrelizumab	*Treponema pallidum* (secondary syphilis)	Serum VDRL 1:2; CSF VDRL negative; chemiluminescent treponemal assay positive; IgM low	Not reported	Not performed (diagnosis by chemiluminescent assay)	Intramuscular benzathine penicillin + IV ceftriaxone; full recovery	Low titers due to B-cell depletion; emphasizes the need for treponemal test; IgM low but IgA/IgG normal
Pipitò et al., 2024 (14)	37-year-old man with MS on ofatumumab	*Treponema pallidum* (secondary syphilis and ocular involvement)	Treponemal and nontreponemal tests repeatedly negative	Not performed	Rectal swab multiplex PCR positive for *T. pallidum* DNA	IV penicillin G (14 days) + single dose IM benzathine penicillin; resolution of rash and ocular symptoms	First reported seronegative syphilis under ofatumumab; PCR required to confirm diagnosis (no seroconversion)
Dop et al., 2013 (15)	66-year-old woman with marginal zone lymphoma on rituximab	*Borrelia burgdorferi* (Lyme neuroborreliosis)	Serum and CSF antibodies negative despite symptoms for 6 weeks	Not applicable	CSF PCR positive for *B. burgdorferi* DNA	IV ceftriaxone (14 days); patient improved	B-cell depletion causes seronegative Lyme; PCR essential
Dahdah et al., 2025 (16)	50-year-old female with follicular lymphoma on obinutuzumab	*Borrelia afzelii* (Lyme neuroborreliosis)	Serologies repeatedly negative	Skin biopsy with *B. afzelii-*specific PCR was positive	CSF NGS positive for *B. afzelii*	IV ceftriaxone (21 days); patient improved	Complete resolution; first report of obinutuzumab-associated seronegative Lyme NB with NGS confirmation

^*a*^ Summary of published cases describing seronegative treponemal or other spirochetal infections occurring in patients treated with anti-CD20 monoclonal antibodies. The table highlights patient characteristics, diagnostic modality, serologic findings, and outcomes. CSF, cerebrospinal fluid; IHC, immunohistochemistry; IV, intravenous; MS, multiple sclerosis; NGS, next-generation sequencing; PCR, polymerase chain reaction; TPPA, *Treponema pallidum* particle agglutination; VDRL, Venereal Disease Research Laboratory.

In our case, the patient had a classic palmoplantar rash and systemic symptoms, yet both RPR and TPPA tests were repeatedly negative. Ongoing diagnostic uncertainty, coupled with awareness of serologic limitations, prompted dermatologists to perform a punch biopsy, which confirmed the diagnosis (refer to Fig. 2 and 3). Because treponemes are abundant in secondary lesions, direct detection methods do not depend on host antibodies and remain reliable even when serology fails. Limitations of our case include lack of baseline immunoglobulin measurements and the absence of CSF analysis, though the clinical presentation did not suggest neurosyphilis.

Several practical recommendations follow. First, clinicians should recognize that negative serology does not reliably exclude infection in patients receiving anti-CD20 therapy. When clinical features suggest syphilis or other infections ordinarily diagnosed serologically, direct pathogen detection (biopsy and PCR) should be pursued promptly. Second, monitoring immunoglobulin levels and B-cell counts can identify patients at high risk for seronegativity; profound hypogammaglobulinemia may warrant immunoglobulin replacement and heightened infection surveillance (5). Third, appreciating that obinutuzumab produces deeper and more sustained B-cell depletion and is associated with higher-grade adverse events and infections should prompt heightened vigilance for seronegative presentations of infections that are typically diagnosed serologically (8). Finally, a multidisciplinary collaboration among infectious disease, dermatology, and oncology specialists can help avoid diagnostic delays.

### Conclusion

Anti-CD20-mediated B-cell depletion can profoundly impair antibody responses to pathogenic spirochetes, rendering serologic diagnosis unreliable in secondary syphilis. Clinicians caring for anti-CD20-treated patients must maintain heightened clinical suspicion and pursue tissue-based or molecular diagnostics when clinical features suggest infection despite negative serology.
